# “Choosing your Own Path”: Patterns of Use of Psychiatric Medication among Individuals with Serious Mental Illness

**DOI:** 10.1007/s10597-025-01465-w

**Published:** 2025-04-21

**Authors:** Maia Asher, David Roe, Rivka Tuval-Mashiach, Ilanit Hasson-Ohayon

**Affiliations:** 1 Department of Psychology, Bar-Ilan University, Ramat Gan, Israel; 2 Department of Community Mental Health, Faculty of Social Welfare and Health Sciences, University of Haifa, Haifa, Israel

**Keywords:** Adherence, Serious mental illness, Psychiatric medication, Patterns of use, Qualitative research, Ideal-type analysis

## Abstract

Most individuals with serious mental illness (SMI) are advised to take psychiatric medication, but about half of them do not take it as prescribed. The binary concepts of “adherence” and “non-adherence” do not seem to capture the actual patterns of medication use. The current study mapped the different patterns of medication use among people with SMI and explored the characteristics of each pattern. Sixteen participants diagnosed with an SMI that used psychiatric medications for at least one year, were interviewed, and data were analyzed using ideal-type analysis. Analysis revealed four patterns of medication use: (1) adherence without doubt; (2) adherence after attempts to stop/reduce; (3) flexible use over time; and (4) tapering off medication. Individuals may shift between these different patterns in their recovery journey, creating the need for tailored therapeutic interventions that adapt to individuals’ evolving needs, beliefs, and preferences.

## Introduction

Psychiatric medication is typically recommended as the primary treatment for serious mental illness (SMI). Serious mental illness refers to a cluster of illnesses including schizophrenia-spectrum disorders, severe bipolar disorder, and severe major depression, all of which share similar functional deficits (Kessler et al., 2003). Although medication is highly recommended for these SMIs, research shows that only about half of the individuals who are prescribed medication adhere to treatment (Semahegn et al., 2020). Throughout history different terms have been used to describe patterns of medication use including compliance, persistence, or concordance (Chakrabarti, 2014; Snowden et al., 2014). Over the last couple of decades, adherence has been the predominant term, mainly replacing compliance, which reflected a one-sided, paternalistic relationship with the health care provider (Vrijens et al., 2012). The word adherence was intended to reflect a patient-centered approach that emphasized patients’ more active and engaged role in treatment decision-making (Myers & Midence,^ 1998^; Zeković et al., 2016).

The World Health Organization (WHO) has defined adherence as the alignment between a person’s behavior, including medication intake, and the healthcare provider’s recommendation (WHO, 2003). A taxonomy introduced after the European consensus meeting in 2009 (ESPACOMP) expanded the definition of adherence to encompass the complete process by which people take medication, including initiation, implementation, and discontinuation (Vrijens et al., 2012). Thus, non-adherence can manifest in various ways including delaying starting, not taking the medication at all, not taking the prescribed dose, or prematurely discontinuing. The dichotomy between “adherence” and “non-adherence” has been criticized in critical discussions and empirical findings (e.g., Velligan et al., 2010; Roe & Davidson, 2017; Zeković et al., 2016), which have led to a shift towards viewing it as a continuum rather than a dichotomous construct, which includes different levels ranging from full adherence to partial and non-adherence (Julius et al., 2009).

Given the complexity of defining adherence, measuring it has also proven quite challenging. The lack of consensus on the way adherence should be measured has led to a lack of standardization and difficulty in drawing conclusions based on data collected in different studies. The aim of systematic reviews and clinical discussions, such as the “Methodological Challenges in Psychiatric Treatment Adherence Research” meeting organized by the National Institute of Mental Health (NIMH), was to summarize adherence assessment tools for psychiatric medication, which can generally be divided into two main categories (Anghel et al., 2019; Julius et al., 2009; Sajatovic et al., 2010; Velligan et al., 2007). The first includes “objective” methods such as pill counts, the use of electronic devices and digital medicine systems, and biological measures (Batra et al., 2017; Knights et al., 2020; Sajatovic et al., 2010). The second category, which is more widely used in SMI, includes “subjective” methods, which usually rely on self or clinician reports. However, some of the disadvantages of these “subjective” instruments are their retrospective nature and their tendency to overestimate adherence, as it is highly influenced by social desirability and conformity; furthermore, some of these instruments measure attitudes toward rather than actual use of medication (Asher et al., 2023; Lam & Fresco, 2015; Lehmann et al. 2014; Sajatovic et al., 2010). To overcome these limitations, it is recommended to use multiple instruments (Anghel et al., 2019). Nonetheless, it is important to note that self or clinician report questionnaires often dichotomize adherence (i.e., adherent vs. non-adherent), or interpret adherence on a continuum (i.e., ranging from high to low adherence on a 5-point scale).

Given the inherent challenges in conceptualizing and measuring adherence, the body of knowledge on actual medication use among people with SMI remains limited, particularly within community settings where there is less contact and monitoring by mental health providers. A recent systematic review of antipsychotic medication use in community settings in the USA revealed significant variability in adherence, ranging from 22 to 67.7% over a six-month period, and from 9 to 71% over a twelve-month period (Martin et al., 2022). High variabilities in adherence patterns were also found in other systematic reviews and meta-analyses focusing on factors influencing adherence (Marrero et al., 2020; Semahegn et al., 2020). In addition, Martin and colleagues (2022) also focused on discontinuation in their review, and found that discontinuation rates varied widely, between 27.4 and 76%, depending on how discontinuation was defined and the length of the follow-up period in the analyzed studies (Martin et al., 2022). Research has also examined the process, experiences, and reasons for discontinuation, mainly focusing on individuals who stopped taking medication and could be labelled as “non-adherent” because of their premature discontinuation of treatment (e.g., Keogh et al., 2022; Morant et al., 2023; Ostrow et al., 2017; Roe et al., 2009). Thus, the considerable variability in both adherence and discontinuation patterns has been highlighted in the current scientific literature, yet there is a lack of in-depth understanding of the diverse modalities of these medication use behaviors, especially among individuals whose adherence status challenges conventional dichotomous classifications of being fully adherent or fully non-adherent, which is likely to be a common phenomenon.

Finally, the way people use their psychiatric medication may be affected by social determinants including employment and educational status, microsystems (e.g., family support), and macrosystems as rehabilitation policies (for example, in Israel the Ministry of Health requires individuals to attend regular psychiatric follow-ups to be eligible for rehabilitation services), and treatment possibilities that are offered to people coping with SMI (Alston et al., 2019; Deng et al., 2022; Higashi et al., 2013; Marrero et al., 2020; Peh et al., 2021; Semahegn et al., 2020; Stentzel et al., 2018). Studies have also emphasized the influence of medical system-related factors, including the availability and quality of medication and health facilities, the quality of health education within the individual’s society, and the government’s provision of various treatment options such as medication-free treatment units (Semahegn et al., 2020; Standal et al., 2021). Therefore, the current study’s aim was to map the main profiles of individuals’ medication use, reflecting their lived experience with medication. Furthermore, we explored whether certain sociodemographic characteristics characterized the different medication use profiles.

## Method

### Study Design and Procedure

Qualitative data from semi-structured interviews analyzed in a previous study by the same researchers (Asher et al., 2023) were used in the current study, alongside the inclusion of a third author (RTM) who has expertise in qualitative research. In addition to the above-mentioned original data analysis, a secondary data analysis (Ruggiano & Perry, 2019) was conducted on the entire dataset for two main reasons: first, to explore a new research question, and second, to explore the dataset using a different analytic approach. Therefore, the current study employed the same procedure framework as the initial study but differed in the data analysis process. Whereas the focus of the previous study was on factors that impact the process of shaping ones’ attitude toward medication and patterns of use, in the current study we aimed to identify and map the actual behavior patterns of medication use. Thematic analysis, the aim of which is to identify similarities and main themes among participants (Stapley et al., 2022), was employed in the previous study. However, given that adherence behavior can manifest in diverse ways, with varied modalities of medication use patterns, an analytic approach that focuses on individual differences was needed. Hence, ideal-type analysis was chosen for secondary analysis as it also emphasizes personal within-case unique experiences and meanings (Stapley et al., 2022). This approach aligns with the need to understand the diverse ways individuals use medication and allowed us to construct typologies that best represent distinctive patterns of psychiatric medication use. By conducting secondary analysis, we were able to maximize the utility of the collected data, enabling the exploration of a new research question and providing insights and a deeper understanding of medication use patterns among individuals with SMI.

The study was approved by Bar-Ilan University’s Committee for Ethical Research (#2021/01). All participants provided written informed consent and were informed of their right to withdraw from the study at any time. Participant anonymity and confidentiality were maintained throughout the study, and any names or identifying information were changed in the interview transcripts.

Participants were recruited over the course of a year from 3 outpatient mental health centers and via social media advertisements (e.g., social groups for people coping with SMI). The recruitment from different settings aimed to achieve a heterogeneous sample that closely reflects the characteristics of individuals with SMI living in community settings. The participants of the current study were the first 16 participants to be recruited to a larger quantitative study focusing on attitudes toward medication and the implications for clinical and personal recovery, and they had agreed to participate in both the quantitative and qualitative studies. Recruitment was carried out until code and meaning saturation were reached (Hennink et al., 2017) in a previous study by the authors (Asher et al., 2023). The recruitment of participants stopped when no new meaningful themes emerged in the thematic analysis, regarding factors that shape attitudes toward and patterns of medication use, which was the focus of that study. As the current research is based on the same dataset, this resulted in a relatively modest sample size. The participants showed interest in the study and were given information from the research team about the research procedure and goals. They then signed a consent form, completed a sociodemographic questionnaire, and were interviewed via the Mini-International Neuropsychiatric Interview (MINI; Sheehan et al., 1998), a structured diagnostic psychiatric interview, to confirm that they had an SMI. Afterwards, participants were interviewed by the first author (MA), a PhD student and a rehabilitation psychologist with experience administering qualitative interviews. The interview was carried out in one session (interview M time = 1 h), except for when participants asked that the session be divided into two meetings (i.e., because the interview was too long). Interviews took place in public settings; or in private, quiet places; or via Zoom due to quarantine restrictions during the COVID-19 pandemic. All interviews were conducted and analyzed in Hebrew, and then specific quotations were translated to English.

### Participants

Participants were 16 individuals (age M = 38.5, SD = 11.32; 9 men) in the post-acute phase of an SMI, living in community settings. Inclusion criteria included a diagnosis of SMI that was severe enough to compromise at least 40% of their functioning and for which they had a defined “psychiatric disability” recognized by the National Insurance Institute (NII). Furthermore, participants had to have used psychiatric medication for at least one year (mainly anti-psychotic meds and mood stabilizers; M = 14.03 years, SD = 8.25, range: 1–26 years). Table 1 presents participants’ sociodemographic characteristics.

**Table 1:** Participants’ sociodemographic data organized by ideal type

	Participant by Group	Age	Education	Marital status	Living environment	Employment status	Medication use time (years)	Type of medication	Diagnosis
Adherence without doubt	P01	27	< 12	Single	SH	SE	6	Antipsychotic	Schizoaffective Disorder
	P03	22	12	Single	Hostel	SE	7	Antipsychotic	Schizoaffective Disorder
	P06	29	12	Single	SH	SE	10	Anticonvulsant	Bipolar Disorder & OCD
Adherence after attempts to stop/ reduce	P02	30	12	Single	Independent	Employed	6.5	Anticonvulsant	Bipolar Disorder
	P05	33	BA	Single	Independent	Employed	15	Antipsychotic	Schizophrenia
	P13	29	MA	Relationship	Independent	Self-employed	7	Antipsychotic	Bipolar Disorder
	P16	48	BA	Relationship	SH	SE	26	Antipsychotic	Schizophrenia
Flexible use over time	P07	48	12	Single	SH	Employed	20	Antidepressant	Major Depression Disorder
	P10	52	BA	Divorced	Independent	Employed	14	Anticonvulsant	Bipolar Disorder
	P12	60	MA	Divorced	Independent	Self-employed	25	Antidepressant & Anticonvulsant	Bipolar Disorder
	P15	43	BA	Married	Independent	Self-employed	24	Antipsychotic	Schizoaffective Disorder
	P08	29	12	Single	Independent	Unemployed	1	Antidepressant	Schizophrenia
	P09	50	12	Married	SH	Employed	26	Antidepressant & Anticonvulsant	Schizoaffective Disorder
Tapering off medication	P04	29	12	Relationship	Independent	Self-employed	6	No use	Schizophrenia
	P14	41	MA	Married	Independent	Self-employed	17	No use	Bipolar Disorder
	P11	47	BA	Married	Independent	Unemployed	14	No use	Schizophrenia

Note: SH = Supported Housing, Hostel = full rehabilitative residential framework, SE = Supported Employment, Anticonvulsant = medications primarily used to treat epilepsy and bipolar disorder including Tegretol, Lemictal and Depalept in the study’s sample

### Instruments

The Mini-International Neuropsychiatric Interview/MINI (Sheehan et al., 1998), a short structured diagnostic interview for DSM-IV psychiatric disorders, was carried out to validate a diagnosis of schizophrenia or schizoaffective disorder, bipolar disorder, or major depression disorder in addition to the information given via self-report.

A semi-structured interview based on the the narrative approach (Josselson, 2013) was conducted. Participants were invited to narrate their personal experiences and the process they underwent with psychiatric medication from the time that the medication was first prescribed for them until the time of the interview. After the open invitation to share their experiences spontaneously, the interview included several questions which aligned with the study aims, for example: “What is your pattern of medication use currently, and what was the process that got you here?”; “How long did it take until your treatment was stabilized to the current one?”; and “Were there times where you stopped taking medication (if so, why)?”

### Data Analysis

Transcribed interviews were analyzed using ideal-type analysis (Gerhardt, 1994), which serves to generate a typology and grouping of similar cases regarding a certain phenomenon. Analysis was based on the seven steps developed by Stapley and colleagues (2022): (1) becoming familiar with the data, (2) case reconstruction, (3) constructing ideal types, (4) identifying “optimal cases,” (5) forming ideal type description, (6) credibility, and (7) making comparisons.

The first step of the analysis was to become familiar with the text, a step that was carried out by reading the text many times in addition to previously analyzing the dataset, enabling the researchers to have an in-depth familiarity with the dataset. The next step involved reading each interview separately and writing a detailed case reconstruction for each participant, a summary of participants’ patterns of use throughout the years and current patterns of use, main characteristics of the narrative, and dilemmas or meaningful views regarding medication raised by the participant.

The third step, constructing the ideal types, was carried out by making ongoing comparisons between the cases, identifying areas of similarity and differences in patterns of use, main characteristics of the participants, their narratives, and their personal views regarding medication. Constructing ideal types was conducted by two judges (MA and DR), psychologists in the mental health field who were highly familiar with the dataset and whose perspectives regarding medication use align with the personal recovery movement. This perspective emphasizes the importance of respecting individuals’ decisions regarding their patterns of use as a personal free choice, which should be listened to and considered within a shared decision-making process with professionals (Oedegaard et al., 2020). The researchers approached the analysis reflexively, and several discussions were held until consensus was reached about the typology which best represented participants’ complex patterns of use and their heterogeneity. All the ideal type groups that emerged were included in the results. The researchers then assigned each participant to an ideal type group, identifying similarities between participants within the group, and distinctiveness from participants in other groups (Stapley et al., 2021).

The fourth and fifth steps were performed simultaneously: “identifying optimal cases” and “forming ideal type descriptions.” For each ideal type, a single case was chosen, referred to as the “optimal case” represented by an illustrative case that best represented the group pattern of use and its main characteristics. Inter-rater reliability for grouping the cases in the ideal types was 87.5% (14 of the 16 cases). Cases that were less unambiguously similar to the set of characteristics of the ideal type were further discussed until the judges reached a consensus.

In the sixth step, the aim was to check credibility and ensure that the data were best represented in the ideal types (Stapley et al., 2022). Thirteen judges masked to the data (M.A. students in clinical psychology) with no previous background in the research topic were randomly assigned to the clean, uncoded transcripts. Each was asked to assign several narratives to an ideal type group, as well as the degree of compatibility with the group on a scale of 1–5. They were not exposed to any information about the participants, nor to the optimal cases which represented the ideal type groups. There was a consensus analysis with a high compatibility degree (i.e., 4–5) of 10 out of the 16 cases. Refinements were made in the ideal type description frame, and one participant classification to an ideal type group was changed. Furthermore, because the sample size was modest, we wanted to make sure that the ideal type groups represented the diversity of patterns of use in a community setting. Therefore, as a further act of validation, we met with D.K, both a mental health service provider and a consumer, with lived experience of service usage, to further discuss the ideal type groupings and main characteristics. She agreed with the typology system and emphasized that individuals can move through ideal type groups depending on their personal recovery processes and changes in illness perceptions. Finally, in the 7th step, we reflected upon similarities within ideal type groups and differences between them in sociodemographic data, presented in Table 1.

## Results

Four ideal type groups emerged from data analysis: (1) Adherence without doubt; (2) Adherence after attempts to stop/reduce; (3) Flexible use over time; and (4) Tapering off medication (see Table 2). Most of the participants’ narratives, in all of the ideal types, began with a period of trial and error with medication use after illness onset.

**Table 2:** Ideal type description frame

Ideal type	Pattern of use	Main characteristics
1) Adherence without doubt	Use medication as prescribed (since symptoms have begun).	• Taking medication does not cause major internal conflict, and there is no internal dialogue on the subject (i.e., passive acceptance). • No significant personal disagreement with the psychiatrists/system. • Unquestioning view that medication is necessary for long-term use to reduce symptoms.
2) Adherence after attempts to stop/reduce	Use medication as prescribed	• There is an internal dialogue about using medication. • Rely on psychiatrists’ opinion, but also consider one’s own personal beliefs. • Based on achieving a certain symptom balance with the use of medication, believe that taking medication is central to recovery.
3) Flexible use over time	Flexibility and lability in patterns of use, while seeking different solutions other than medication.	• A continuous search for alternative treatment options other than medication, to cope with the illness. A complex process of a search which includes fluctuations in patterns of use. • The alternative solutions often match participants’ personal sets of beliefs and values. • The search process usually includes times of struggle (including disagreements with psychiatrists/system about treatment), as well as a feeling of empowerment.
4) Tapering off medication	Individuals who have withdrawn completely from medication.	• People who have stopped taking medication altogether, and went through a withdrawal process. • A general negative attitude toward medication, and a belief that medication is harmful when used permanently and/or over the long-term. These individuals are critical of the mental health system and psychiatry. • The feeling of succeeding in withdrawing from medication is presented as a main and significant aspect in their view of their recovery process.

The following section includes a detailed description of each of the four ideal types, which are demonstrated by an illustrative case that most closely represented the group characteristics.

### Adherence without doubt

Three participants represented this profile that clustered together people who used medication as prescribed and showed no desire or motivation to change their pattern of use. If there were times when they didn’t take the medication, it was unintentional, due to a misunderstanding of the way the medication should be used (e.g., on an everyday basis), or they had forgotten to take it. Their adherence levels were described as consistently high, and they were not preoccupied with the issue of psychiatric medication as they had adopted the psychiatrists’ recommendations without experiencing any conflict or dilemma.

#### Illustrative Case: Daniel

Daniel was a 22-year-old man who lived in a hostel in the community (i.e., full rehabilitative residential framework) and had been diagnosed with schizoaffective disorder at the age of 18. Daniel described a period during which his psychiatrist adjusted his medication a few times until finding the right “cocktail” and dosage that would help reduce symptoms but didn’t have too many side effects. Daniel said: *“The medication helps (silence). Although it took some time to get used to it.”* He was always fully adherent to the medication and followed the psychiatrists’ instructions; furthermore, taking the medication did not evoke any internal dilemmas: *“The medication reduces my symptoms; I’m more balanced with it and I take it every day… and if I didn’t take it*,* I wouldn’t feel better.”*

### Adherence after attempts to stop/reduce

Four participants represented this profile that clustered together people who negotiated possible modifications with their treating psychiatrist and eventually made changes in the type and/or dosage of their medication, reaching a point that felt right for them; they then fully adhered to the medication prescribed. These individuals had undergone a period during which they felt “something was off” due to their use of medication, whether it was side effects, feeling not themselves, or whether taking medication daily did not align with their personal perceptions of themselves or their identities. Modifications in their patterns of use were made as a result of their consultations; they took under consideration both the psychiatrists’ recommendation as well as their own feelings and thoughts about the effects of medication on them. After changes or adaptations were made in their medication use, they developed a generally positive attitude toward medication.

#### Illustrative Case: Anna

Anna was a 30-year-old woman, lived by herself, and worked as a mental health service provider with lived experience. She was also a mental health service consumer, having been diagnosed with bipolar disorder at the age of 24. As can be seen in the example below, Anna described the first years after illness onset as a period during which she didn’t feel like herself, suffered from many side effects (from the medication), felt incompetent, and had low self-efficacy:

*The medication affected me on so many levels*,* at work*,* and had an effect on my feelings of competence. I didn’t recognize myself anymore. Where is that woman who used to wake up for work every day? My identity had changed*,* something was broken. A lot of it had to do with the medications…When I left the hospital*,* no one told me that there were other possibilities*,* that I could feel different. I just took the medications*,* although they really harmed my quality of life… The whole issue of what it means to take medication was not discussed; you just take them at 08:00 am and 08:00 pm*,* and that’s about it.*

Anna was concerned with the issue of medication, feeling that the medication wasn’t quite right for her. Only after changing her treating psychiatrist and obtaining a good therapeutic alliance with a new one did she begin reducing her use of medication, and this reduction resulted in a significant positive change in her attitude toward medication: “*Due to the reduction*,* I don’t feel the overload from the medication anymore. These days it’s just a small pill that keeps me in check and supports my recovery.”*

### Flexible use over time

Six participants represented the third profile that clustered together people who were characterized by sporadic and labile patterns of use. These individuals made different kinds of attempts to change medications and dosages, which included attempts to reduce or withdraw, including taking medications only when symptoms flared up. They sought personal solutions and alternatives to the common treatment guidelines, which better matched their sets of beliefs or values, such as alternative medicine or spirituality. The participants in this cluster usually tended to reject biogenetic explanations for psychiatric disorders and held more diverse ones, including psychosocial, psychological, and spiritual explanations.

#### Illustrative Case: Lili

Lili was a 52-year-old woman, divorced, lived with her two daughters, and worked as a teacher. She was diagnosed with bipolar disorder at the age of 37, after experiencing a traumatic event in her family. Lili described having many struggles. She had tried to manage her symptoms, had extensive feelings of shame and internalized stigma, and felt powerless in the mental health system: “*The diagnosis*,* all the medication*,* and so much shame. I was so afraid that people would know I was mentally ill. I felt so much stigma*,* and I gave a lot gave a lot of authority to the things my psychiatrists said.*” She made several attempts to reduce her medication, and one attempt to stop the medication altogether resulted in a relapse. As time progressed, her sense of agency increased, and she began to search for other solutions. She read many books and articles about psychiatric medication and its alternatives, she went to peer group meetings that included people who had tapered their medication use, and she began psychotherapy, where she felt seen and connected. She started to get in touch with her strengths and to believe that she could find a better path for herself:

*Over the past few years*,* I have been strengthening myself again*,* my abilities and capacities and inner faith. I work on it all the time; it’s an endless process… Today I manage my illness. I decide who my therapists will be*,* how I want my treatment to look. I’m in charge and I have the power. Nobody else can tell me what to do. These days I decide things after consultations*,* reading a lot*,* investigating the risks and benefits*,* going to lectures and peer group meetings. For example*,* I am currently tapering*,* and it’s possible that this is a mistake and that I’ve made the wrong decisions*,* but they are my decisions to make. I believe that the power for change is always in the hands of the person. We should never*,* ever attribute the possibility for change only to the medication.*

### Tapering off medication

Three participants represented the fourth profile that clustered together people who chose to withdraw completely from medication. They described difficult experiences with medication such as side effects, withdrawal symptoms, and the feeling that the medications changed their perceptions of themselves, their identity, and significantly harmed their recovery process. They held a negative attitude toward medication and had doubts about the mental health system’s ability to help them. They identified themselves in their narratives as non-conformists, given that the process of permanent withdrawal required a lot of willpower, a significant family/spouse support, and belief that it was possible. The fact that they succeeded in tapering from their medication greatly empowered them and gave new meaning to their personal narratives.

#### Illustrative Case: John

John was a 47-year-old man who lived with his wife and children. At the time of the interview, he was unemployed, but he had a long history of working in the high-tech industry. He was diagnosed with schizoaffective disorder at the age of 30 after becoming a father and losing his own father, and he stopped taking his medication three years prior to the interview. John shared his belief that people gave too much power to their doctors and had too much confidence in them, instead of “choosing their own paths” in their personal recovery processes:

*If you do not question your doctor’s instructions*,* you become dependent. When you become dependent in this way*,* you hinder your recovery*,* which is a personal process… Imagine you are riding your bicycle and you want to try riding without hands. A lot of times when you try this you will fall*,* but you will also get up*,* and it is the same thing with tapering. I believe that it’s the person’s choice: how he wants to ride his bike and live his life. I listen to the doctors’ opinions*,* but I’m the one in charge of my quality of life and my mental health. It’s my choice to make.*

During the years of coping with his illness, John had reduced or stopped taking his medication several times, returning to its use only at times of crisis or relapse. At the age of 44, three years prior to the interview, he felt that he was in a stable place in his life and had managed to complete his process of full withdrawal from medications. He shared his negative attitude toward medication and the pressure he felt from those around him to take medication:

*Since the start*,* I didn’t like the medication. I felt that it damaged my self-confidence. I didn’t want to just accept that I needed it; I wanted to manage on my own. It did do some good and helped when my symptoms were very strong. But you lose the feeling of being alive; you live at a much lower intensity. Those in your environment are usually happy about this*,* but you feel that the medication limits and restrains you.*

### Sociodemographic Data and Medication Use

Participants’ detailed sociodemographic data, divided into ideal type groups, are presented in Table 1. Two observations should be noted, as emerged in the current study’s sample. First, all participants in the “adherence without doubt” ideal type lived in a supported housing environment (i.e., hostel which applies a full residential framework or supported housing) and participated in a supported employment program. Such settings entail extensive support, professional assistance, and regular contact with psychiatric community services, settings which may also encourage full adherence as the preferred, or sometimes as the only possible approach for treatment. In all other types of medication use, most participants lived independently in the community, were employed or self-employed, and did not require extensive support services. Second, all the participants in the “tapering off medication” ideal type were in a relationship or married, whereas participants in the other ideal types had varied marital statuses.

## Discussion

The present study reveals a typology identifying distinct types of patterns of medication use, among individuals with SMI living in community settings. The findings uncovered four primary profiles of use: adherence without doubt, adherence after attempts to stop/reduce, flexible use over time, and tapering off medication. These patterns challenge the notion of adherence as a simplistic binary concept, shedding light on the diverse approaches and ways individuals navigate their treatment choices, moving beyond a singular view of adherence. It is important to note that choosing one’s patterns of medication use represents an ongoing process people face when coping with SMI (Asher et al., 2023). Therefore, the classification of a participant to an ideal-type group refers to a single point in time during a dynamic and often long trajectory. This dynamic nature of medication use is distinct from common typologies in psychology research which usually represent more deterministic constructs.

The first profile, “adherence without doubt,” represented participants who were fully adherent to medication use without conflict. However, the participants in this group showed higher rates of utilizing supported housing and employment services, indicating lower functional recovery and a greater need for support (Thomas et al., 2018). Furthermore, these individuals may also have lower clinical recovery, characterized by higher symptom levels, which could contribute to their adherence without attempts to modify their medication use, as changes such as reduction or discontinuation might present significant challenges and increase the risk of relapse (Moncrieff et al., 2023). These characteristics are likely to reflect increased contact with and dependence on the mental health system and/or caregivers, possibly resulting in internalizing the tendency to encourage full adherence. Furthermore, as some services are explicitly or implicitly conditioned on adherence, individuals consuming these services may adhere without questioning, in order to avoid cognitive dissonance. In Israel’s mental health system, for example, to be eligible for the kinds of rehabilitation services referred to herein, the person must participate in regular psychiatric follow-up visits (Knesset Research and Information Center, 2022). Thus, it is possible that individuals in the “adherence without doubt” group do not engage in reflecting on treatment choices or personal preferences/beliefs, due to policies and restrictions of the psychiatric services they consume. Moreover, it is also possible they internalized the idea that taking medication is the obvious or only choice.

The second profile, “adherence after attempts to stop/reduce,” shares the first profile’s actual behavior of being fully adherent. However, there were two main distinctions between these two profiles. In the second profile there were lower rates of using rehabilitation services, and the individuals in this group had more independent work and housing solutions. In addition, and importantly, they showed an internal ongoing dialogue about medication use and expressed self-efficacy through making their own choice after actually having tried to stop or reduce their medication use. These distinctions contribute to the body of qualitative literature showing the heterogeneity among people with SMI who are adherent in different dimensions. For example, although adherence to psychiatric medication has a positive outcome in reducing symptoms, in some cases it might have adverse effects that harm the person’s functional abilities and affect the person’s agency and sense of self (see systematic reviews and meta-analysis of Bjornestad et al., 2019; and Thompson et al., 2020).

The third profile, “flexible use over time,” best represents the limitations of traditional measurement methods of adherence (see Sajatovic et al., 2010 for review), as these methods cannot properly measure the different ways people actually use their medication. The “flexible use over time” pattern was also demonstrated in a study that followed outpatients’ medication adherence patterns, in which it was found that the typical patient took medication for 15% of the 180-day period, with notable gaps in persistence (i.e., median of Continuous Measure of Medication Acquisition = 0.15; Ramamurthy et al., 2021). The individuals within this group can’t simply be labeled adherent or non-adherent, and obtaining a single score for adherence rate, as the continuum approach would suggest, does not reflect the complexity of their medication use. Their pattern of use varies depending on their current struggles, the progression of their illness, and evolving recovery processes.

An exploration of the fourth profile, “tapering off medication,” which represents those individuals who have stopped taking medication, contributes to the body of research that challenges the idea that medication adherence is the only rational choice (e.g., Geyt et al., 2017; Grover et al., 2014; Katz et al., 2019). Recent studies have indicated that for some individuals, discontinuation of medication can have positive long-term outcomes, especially when considering measures beyond clinical symptoms. For instance, a randomized controlled trial that assessed outcomes of medication discontinuation versus maintenance over a two-year period among individuals with SMI (Moncrieff et al., 2023) showed that although a higher percentage of individuals in the reduction group experienced a symptom relapse (54.4% compared to 30.8%), there were no significant differences in social functioning between the groups, and those in the reduction group reported fewer medication side effects (Moncrieff et al., 2023; Morant et al., 2023). In the present study, individuals who chose to discontinue their medication emphasized the significance and meaning of the withdrawal process and their belief that it enhanced their recovery. This finding aligns with findings from other qualitative studies, indicating that discontinuation from medication can positively affect one’s sense of self, be empowering, and improve peoples’ self-management of the illness and independent living (Morant et al., 2023; Katz et al., 2019). Improvement in these areas of experience and functioning is crucial in the process of personal recovery (Van Weeghel et al., 2019). It should also be noted that the sociodemographic data (see Table 1) revealed that all participants in the current study’s “tapering off medication” profile were in relationships or married, suggesting that support can be meaningful in withdrawal processes, as reflected in participants’ narratives who marked the importance of family and spousal support. Although prior research has consistently mentioned the positive relation between family/social support and high adherence levels (Alston et al., 2019; DiMatteo, 2004; Marrero et al., 2020), the current small sample study suggests that family and spousal support can be also helpful in stopping medication use.

The different characteristics of the profiles hold possible implications for policy and practice. Policymakers should reconsider conditioning mental health services on full medication adherence, and services should focus on providing comprehensive support tailored to individuals’ needs while simultaneously encouraging personal reflection and choice. Also, services should aim to conduct more educational programs and workshops that offer extensive information on medication use in SMI, covering alternative treatment options in community settings and self-management techniques. Furthermore, mental health providers should value and foster individuals’ self-efficacy, empowerment, and active participation in treatment decisions; treatment options and their influence on the person should be discussed (Conneely et al., 2024; Jaiswal et al., 2020).

The current study had several limitations. First, it should be noted that it is advised for ideal-type analysis to consist of a larger sample size, typically 30 cases (Stapley et al., 2022), and caution should be taken when generalizing the results to a broader context. For our study, we were able to recruit 16 participants within a twelve-month timeframe, signifying a modest sample size. Difficulties in recruitment were related to the inclusion criteria (see Participants section) and to the influence of COVID-19 imposed restraints. Moreover, as recruitment was carried out until saturation was reached in a previous study with a related topic (see Study design and procedure), it increased the possibility we missed other meaningful patterns of use, that were not reflected in the study’s sample. Second, the focus in community settings, with the aim of representing a variety of individuals with SMI in different stages of the illness, made it difficult to reach certain potential participants. It was especially challenging to recruit participants from hostels, where people didn’t express a special interest in the subject and, in a few cases, management expressed concerns about talking with tenants regarding the issue of medication. Therefore, the study sample may have been biased towards people who tended to be more independent and motivated to share their experiences. In addition, this study did not addressed factors that can explain specific profiles, such as insight into the illness in the case of non-adherence. Last, it is possible that the different medication uses patterns have also been influenced by the underlying heterogeneity of the study’s sample (see Table 1). To address some of these limitations, we added to the analysis process comprehensive discussions with a mental health service provider and consumer who had lived experience with SMI (D.K), and we also performed a consensus analysis (see the sixth step in the data analysis), to ensure we best represented the profiles of use within community settings. The insights gained from the discussions with D.K significantly enriched our understanding regarding the research findings. Future studies should consider involving individuals with lived experience in all stages in the research process, as their involvement enhances the relevance and applicability of findings, ensuring alignment with real-world recovery experiences (Stratford et al., 2016).

With these limitations in mind, four main profiles of different patterns of medication use were identified in the current study, contributing to a more nuanced understanding of how individuals with SMI actually use their medication. Three key implications emerged. First, the findings highlight the need to reconsider the term “adherence,” commonly used in theory, research, and clinical practice. Previous research has already suggested that the terms “adherence” and “non-adherence” fail to capture the complexity of decisional aspects regarding medication use (Roe & Davidson, 2017), and we would thus advise using the term “patterns of use,” which represents the diverse ways in which individuals interact with psychiatric medications over time. Considering our findings of different patterns of use, it might be also useful to consider expanding the flexibility and range of prescribing of the medication. Second, it is recommended that future research combine current measurement methods of adherence with qualitative inquiries, aligning more congruently with the consumer’s subjective experience, as offered in the current study. The four profiles of medication use that emerged in this study may help, if validated and supported by future large-scale research, to capture the diversity and complexity of patterns of use beyond the continuum approach. Third, it is essential to acknowledge that individuals may traverse between these ideal types at different time points in their process of coping with SMI and personal recovery processes. Future research should aim to better capture possible modifications in patterns of use in a longitudinal design, identifying factors that influence these changes and their impact on the individual’s well-being and different recovery outcomes aside from clinical symptoms. The recognition of these dynamic shifts highlights the importance of therapeutic interventions that remain attuned to the evolving patterns of medication use. As individuals progress through their recovery, it becomes crucial to identify their currently dominant pattern, thereby facilitating early detection and relapse prevention, as well as offering tailored interventions that align with the person’s needs. In doing so, shared decision-making, the exploration of possibilities, and the weighing of pros and cons of medication use would all be enhanced.
